# Use of healthcare services in the region of origin among patients with an immigrant background in Denmark: a qualitative study of the motives

**DOI:** 10.1186/s12913-016-1346-1

**Published:** 2016-03-21

**Authors:** Nicoline Lokdam, Maria Kristiansen, Line Neerup Handlos, Marie Norredam

**Affiliations:** Section of Immigrant Medicine, Department of Infectious Diseases, Copenhagen University Hospital, Hvidovre, Denmark; Research Centre for Migration, Ethnicity and Health, Institute of Public Health, University of Copenhagen, Oster Farimagsgade 5, 1014 Copenhagen K, Denmark

**Keywords:** Cross-border healthcare use, Immigrants, Denmark, Mobile patient

## Abstract

**Background:**

In Denmark, immigrants have been found to have a higher use of healthcare services abroad. Since this use may have an impact on both the individual patient and the healthcare system in the country of residence, research into underlying reasons is of increasing relevance. This study therefore investigates what motives patients with an immigrant background have for seeking healthcare services in their region of origin.

**Methods:**

The study was based on 10 semi-structured interviews with 10 patients who had an immigrant background, primarily originating from Turkey and the Middle East, recruited at a clinic of immigrant medicine in Denmark. The interviews were analysed thematically to elucidate motives for seeking healthcare services abroad, with focus on identifying push and pull factors.

**Results:**

Four motives for seeking healthcare in the region of origin were salient in the material: the perception of availability, in terms of quantity and access; familiarity, conceptualised as feeling comfortable within the healthcare system; perception of quality of services; and finally, the perceived need for a second opinion. All motives emerged simultaneously as push factors, motivating immigrants to explore healthcare services abroad, and pull factors, attracting them to their country of origin. Affordability did not emerge as an independent motive but influenced the other factors.

**Conclusion:**

The use of healthcare services abroad by patients with an immigrant background constitutes active health-seeking behaviours shaped by a range of factors perceived to be limiting access to high-quality services in Denmark. Further research, including quantitative studies, should be initiated to investigate the importance of these motives among larger, more diverse immigrant groups, consequences for treatment regimes, and the healthcare professionals’ perspective on the use of healthcare in the region of origin among immigrant patients.

**Electronic supplementary material:**

The online version of this article (doi:10.1186/s12913-016-1346-1) contains supplementary material, which is available to authorized users.

## Background

Research on the use of healthcare services across national borders is an area of increasing relevance in public health due to globalisation processes and increased mobility of patients. This study investigates which motives immigrants in Denmark have for using healthcare services in their region of origin. We find it relevant to analyse these motives in the context of push and pull factors [1], since factors shaping health-seeking processes are likely to appear both in the country of residence and in the region of origin.

In Denmark, anyone with a valid residence permit and who is registered in the civil registry has the right to access all public healthcare services [2]. This includes access to general practitioners (GP) and in- and outpatient hospital-based care. Within this healthcare system, the GP functions as a “gatekeeper” as patients need referral to specialized healthcare services in non-acute situations. The Danish healthcare system is financed through taxation, and therefore, most healthcare services are free of charge at access.

Migration patterns have led to increased ethnic diversity in Denmark, like in most European countries, with approximately 10 % of the Danish population having an immigrant background [3]. The majority of immigrants living in Denmark were born in non-Western countries, and the largest ethnic groups among these are from Turkey, Iraq, and Bosnia and Herzegovina [4]. Being a migrant is associated with different potential health risks due to exposure to risk factors occurring before, during, and after migration [5]. In addition health behaviours and illness perceptions are shaped by a mixture of culture, ethnicity, socioeconomic background and acculturation processes, thus leading to great diversity in health-seeking behaviours among and even within different migrant groups [6–12]. In general, immigrants from non-Western countries are more likely to belong to low socioeconomic position groups compared with local-born individuals [4]. This makes immigrants, especially women, a particularly vulnerable group in terms of access to high-quality healthcare as well as possibilities for engaging in health promotion activities [13]. Besides socioeconomic obstacles, studies of access to healthcare for immigrants show that some of the most common barriers to healthcare utilisation for immigrants in their country of residence are related to language, communication, and mistrust with the healthcare system; different expectations between immigrant and healthcare providers about medical procedures; and finally, experienced prejudicial behaviour from the side of healthcare providers [14, 15]. These barriers may lead to a reduced or inappropriate use of healthcare services in the country of residence, and consequently seeking healthcare services in the region of origin may be a way for immigrants to cope with the barriers.

The cross-border use of healthcare services among immigrants in Denmark has been documented by Nielsen et al. (2012), who found that Turkish immigrants had a much higher use of somatic healthcare services in a foreign country than ethnic Danes, adjusted for health status, socio-demographic factors, and socio-economic factors [16]. Results from the Netherlands have also shown utilisation of cross-border healthcare in the country of origin, especially among people with Turkish origin [17]. The term “medical tourism” has been used to describe patterns of healthcare use characterized by patients seeking healthcare in a country which is not their country of residency [18, 19]. While medical tourism tend to be used for wider groups of patients, including ethnic majority populations seeking healthcare abroad, and behaviour thus sharing some underlying factors with patients of migrant origin, the present study focuses specifically on the processes and characteristics tied to migrant groups that often face specific challenges in accessing healthcare. For the patients, healthcare providers, and society as a whole, there can be various consequences when patients seek healthcare services abroad, though the evidence underlying these possible outcomes is lacking [20]. Negative outcomes may include (1) disruption of the continuity of care, especially among patients with chronic illness; (2) lack of follow-up and rehabilitation; (3) risk of poor quality of drugs; and (4) the risk of double treatment and/or medication. All of these issues may imply negative effects to the patients’ health and unnecessary costs for the healthcare system. On the positive side, patients may receive faster and cheaper treatment than possible in Denmark and perhaps appreciate it more, if it takes place in a familiar environment [21].

Other studies have focused on immigrants’ use of healthcare services in their country or region of origin [7, 17, 22–25]. One study, carried out by Migge and Gilmartin (2011), used Glinos et al.’s typology [18] on immigrant patients in Ireland, focusing on the four motivations: affordability, availability of care, perceived quality, and familiarity. The study found that affordability and perception of quality were the two main reasons for seeking healthcare abroad among their study population of 60 immigrants with various ethnic and social backgrounds [24]. Sekercan et al. (2014) investigated healthcare consumption among ethnic minority people living in the Netherlands in their country of origin using survey data. They found that the most important motivations were health status, dissatisfaction with care in the country of residence, and seeking a second opinion [17]. To our knowledge, no other studies have investigated what motivations immigrants have for using healthcare services in their region of origin in a European context and none in a Scandinavian. We therefore found it relevant to get a better understanding of the use of healthcare services in the region of origin among patients with an immigrant background living in Denmark. Through a qualitative study, we elucidate factors motivating patients with an immigrant background to use healthcare services in their region of origin.

## Method

### Setting

This interview study was carried out at the Department of Infectious Diseases at a major hospital situated near Copenhagen. The clinic where the study took place is particularly assigned to secure efficient examination, treatment, and rehabilitation of vulnerable patients with an immigrant background. The patients referred to the clinic are primarily from non-Western countries, especially Turkey, Iraq, and Lebanon. Most of the patients are female and between 35 and 65 years of age. The clinic currently has 145 patients.

### Recruitment and study population

Fieldwork was conducted at the clinic that served as the setting for recruitment and interviews. The participants for the study were recruited from the administrative register of patients referred to the clinic by a random selection process according to month of birth. Since female patients are overrepresented at the clinic, this was also the case in our random selection, where only three patients were men. A total of 40 patients were randomly selected and screened by the healthcare professionals at the clinic, which led to the exclusion of 12 patients due to concerns about their poor health status or unavailability. A total of 28 patients were invited to participate by mail and subsequently contacted by phone. Among these, 14 agreed to participate; however, four did not turn up for the scheduled interview. Participants were given the option to be interviewed in their own home, but all preferred to be interviewed at the clinic, mostly as an extension of an appointment at the clinic or another department of the hospital. In total, 10 interviews were carried out during September and October 2014. In five of the interviews, the participants preferred using an interpreter. In these cases, we used video interpretation. Video interpretation is carried out using a special phone with a video screen attached to it and the interpreter is interpreting through the video screen, while physically situated at a translating bureau. This method is often used at the clinic and those participants who were in need of interpretation were therefore familiar with the use of video interpretation.

### Interviews

The interviews were conducted using a semi-structured interview guide [26], with questions arranged into themes (see Additional file 1). The themes were: Participants’ background, perception of health status and needs, communication, utilisation of healthcare services in the region of origin, and consequences when returning to Denmark after receiving healthcare services abroad. The themes in the interview guide were inspired by an informal review of the literature related to cross-border healthcare use among immigrants, especially the typology developed by Glinos et al. [18]. Interviews took place at the clinic and were conducted by a female interviewer. If the participant had attended a consultation at the clinic the same day, the interview setting would be in a different room. The interviewer wore her normal clothes and offered hot or cold beverages in order to create a relaxed atmosphere. The interviews lasted from 15 to 94 min and were audio taped if the participants agreed when giving their informed consent. After each interview, the participants were given a small present. This was not revealed to the participants until after the interview had been completed.

### Analysis

The interviews were transcribed and a manual thematic analysis was conducted, starting with a close reading of the transcribed interviews to identify the main factors that shaped the use of healthcare services abroad. After this first reading, the themes ‘familiarity,’ ‘quality,’ and ‘availability regarding time and access’ were identified as factors shaping seeking healthcare services in the region of origin. The feeling of familiarity was expressed in statements related to perceived similarities regarding language, culture, and perception of health and treatment in the region of origin. We use the term ‘familiarity’ as a notion for whether the participants felt they were familiar with the system or situation, which is strongly influenced by language barriers, literacy, and social network. After the initial reading and thematization, all interviews were read again to identify ‘meaning units’ or quotes that could be of relevance to the research question. When all quotes were picked out, they were sorted into which theme they could exemplify. When an important quote did not fit into an existing theme, a new theme was made. Through this process, the final theme entitled ‘second opinion’ emerged. In the end, the different categories were condensed, and illustrative quotes were chosen with emphasis on illustrating the diversity as well as commonalities in responses given to each question. The analysis was discussed with co-authors and presented to an interdisciplinary team of researchers to ensure reflexivity in the analytical process.

### Ethics and participation

The study was approved by the Danish Data Protection Agency through the Office of Data Security in the Capital Region of Denmark (No. AHH-2014-0008). In Denmark, approval from the ethics committees is not required for interview studies. Before the interviews started, the participants were introduced to the purpose of the interview and their right to withdraw from the study at any time, and they were asked to sign a declaration of informed consent before participating. The statement of informed consent was written in Danish. Therefore, those participants who preferred having an interpreter for the interview had the statement of informed consent translated orally by the translator before the interview began.

## Results

The participants were women who were 41 to 58 years of age (mean age = 49 years). The most common country of origin was Turkey, followed by Iraq. All participants had lived in Denmark at least 5 years and had residence permit. None of the women were currently employed, but most of them had been working in Denmark. Those who had been working stopped working due to chronic illness and pain. None of the participants had obtained any education in Denmark besides Danish language classes. The participants came to the clinic with various kinds of illnesses and disabilities; the most common reasons were chronic musculoskeletal pain or stomach pain. An overview of the characteristics of the 10 participants is shown in Table 1.

**Table 1:** Study population: Characteristics of the *participants* and their use of healthcare service in region of origin

Participants	Age	Country of birth	Experiences with healthcare services in region of origin
1	40–44 years	Morocco	No personal experiences, but experiences through relatives
2	40–44 years	Turkey	Examination of stomach region
			Medication
3	45–49 years	Iraq	Medication and medical cream
4	45–49 years	Turkey	MRI^a^ of back region
			Acute treatment after fainting
5	55–59 years	Turkey	Treatment after acute illness including tests and MRI^a^
6	50–54 years	Iraq	Medicine and injections against musculoskeletal pain in her legs
7	55–59 years	Turkey	No personal experiences, but experiences through relatives
8	45–49 years	Turkey	Medication and examination after pain in stomach region
9	50–54 years	Somalia	No experiences, neither personal or through relatives
10	45–49 years	Lebanon	Injections against musculoskeletal pain in back region

^a^ *MRI* magnetic resonance imaging

Of the 10 participants who were interviewed, seven had personally utilized healthcare in a country in their region of origin. The term ‘region of origin’ has been chosen instead of country of origin because we wanted to explore the participants’ use of healthcare services in a country they felt familiar with and not only their country of origin. The participants who had experienced utilizing healthcare services in their region of origin often mentioned receiving different kinds of medicine or injections; two had received magnetic resonance imaging (MRI). Among the three participants who had not utilized any kind of healthcare services in their region of origin themselves, two of them knew people, either friends or family, who had. In most cases, the healthcare services were provided in the country of origin, whereas one participant had utilized healthcare services in three different countries. Fainting and chronic pain in the back, leg, or stomach region were the most common symptoms or illnesses for which the participants received examination and treatment. The participants had utilized both planned and unplanned healthcare services. Some described how they sought acute treatment during a vacation. Others had planned trips to their home country with the sole purpose of receiving a scheduled treatment at a hospital.

### Reasons for seeking healthcare in the region of origin

Different factors affecting the use of healthcare abroad emerged from the data. The common factors are presented and discussed below, and depicted in Table 2.

**Table 2:** Push and pull factors: Motives for using healthcare services in the region of origin

Motives	Availability	Perception of quality	Need for a second opinion	Familiarity
Push factors	Long waiting lists for specialised care	Long waiting lists for specialised care	Feeling uninformed	Communication barriers
	Restricted access to specialised care	Restrained access to specialised care	Lack of trust between patient and health professionals	Different expectations among patients and professionals
			Little familiarity with healthcare system in the country of origin	
			Lack of further options for treatment	
Pull factors	Perceived easy access: • No waiting lists • No need for referral	Fast and efficient treatment	Familiarity with the healthcare system in the country of origin	Feeling comfortable and secure in the healthcare system
		Specialised doctors	Perception of quality as better	No barriers in communication or language
			Searching for further options	

### Availability

The availability, either in the sense of quantity (e.g., waiting time related to treatment) or in access (e.g., whether you need a doctoral referral before you can get certain tests and treatments), was regarded as being different when the participants compared the healthcare system in Denmark with the healthcare system in their region of origin. More specifically, the participants described difference in efficiency of the doctors in Denmark compared to the region of origin; the doctors in the region of origin were perceived as being more efficient than the doctors in Denmark:

*‘In Denmark, the doctors are much slower. In Turkey, they are much faster. You will almost get an answer the same day. If you need more things done like MRI or blood tests, it will only take a couple of days more before you get a result.’ – Participant no. 8.*

To the participants, the main advantage of seeking healthcare services in the region of origin instead of Denmark seemed to be the aspect of time and availability. The perception of unavailability of treatment in Denmark therefore was an important push factor. As a participant said,

*‘You can’t just go to the doctor. Here* [in Denmark], *you have to make an appointment, so it takes time. Sometimes with these specialised doctors, it can take months. But in Turkey, if a doctor gives you a referral, you can go to the other* [specialised] *doctor within a couple of hours.’ – Participant no. 4.*

Hence, seeking healthcare services in the country of origin was regarded as a way to avoid the long waiting lists and waiting times that the participants experienced in Denmark.

### Perceived quality

The perception of quality of the healthcare services utilized by the participants, seemed to be influenced by how quickly they were able to receive examinations, answers and treatments from the health care professionals when seeking healthcare services. The effect of the treatment, in particular whether it reduced their pain immediately, was another important element. All the participants in this study suffered from chronic disease(s) and/or chronic pain. Almost all of them explained how they had received different kinds of examinations and treatments in Denmark and described how their doctors could not do much to relieve their pain or symptoms. To some participants, the feeling of having pain and illness that the doctors could not explain or treat also led to a feeling of being ‘thrown’ from one doctor to another, without getting any useful help:

*‘They can’t treat me, so all they do is talk and give me a new consultation in two weeks’ time to see if anything’s changed (…) And I know my illness is chronic. There is no cure, but every time I go to my doctor, she just sends me off to some other doctor. She doesn’t do anything herself. She doesn’t even try.’ – Participant no. 4.*

Some participants explained that they did not receive sufficient explanation related to their illness from the doctor, but instead were turned away without receiving a satisfying examination or treatment. This is different from the perception of how their diseases were handled in the healthcare system in their region of origin. The participants who had tried utilising healthcare services in their region of origin described their experiences with much focus on the perceived efficiency and thoroughness of the examinations and treatments they had received:

*‘If you’re at the hospital there* [in Turkey]*, they just examine all your problems at once. They take blood test, urine tests, and all kinds of tests. They will find the problem. They will not just tell you to go home and come back in 14 days.’ – Participant no. 4.*

Other participants were more critical toward the use of medication in their country of origin. One woman expressed a concern with the methods she had experienced when her young daughter needed healthcare during a holiday in Lebanon:

*‘In Lebanon, they give you many different medications (…) When my young daughter was ill, they changed her medication every week, so I was worried, because in Denmark, they don’t give you much medication, but he* [the Lebanese doctor] *explained that they were just trying different medications to see if it was one thing or another.’ – Participant no. 10.*

### Need for second opinion

The opportunity to seek healthcare services in the region of origin seemed to some participants as a further possibility when the Danish healthcare system could not offer any cure or suitable treatment to their chronic conditions. In these cases, seeking healthcare services abroad was not contradicting to getting healthcare in Denmark; it was a part of the participants search for any useful treatment:

*‘If you’re in pain, you have to look at different places to find out how you can get better. It’s not because they think Denmark doesn’t give a good treatment. No, that’s not it. But people search for possible ways of help.’ – Participants no. 10.*

Some of the participants described the search for treatment in their region of origin as the last chance for finding a helpful treatment. One participant described this with the example of her friend who was severely ill and had been told by the doctors in Denmark that she would die:

*‘They can’t help her anymore, so she just says, ‘I have to try in Turkey’, because she is so sick. Very sick people go because they say, ‘we should also try our last chance’, right?’ – Participant no. 7.*

These participants did not necessarily describe the healthcare services in their region of origin as more efficient or of better quality but simply as a place where they could obtain a second opinion related to their disease. One participant had received many kinds of treatments in Iraq, Syria and Lebanon, mostly injections in her knees, which had a good effect on her pain, but the effect was always temporary, and the pain returned after a while when she returned to Denmark:

*‘I try everything. Every time I go abroad to get a treatment, I feel much better when I come back. I don’t have pain. But then after a while, the pain comes back and I get depressed. I can’t stand it anymore.’ – Participant no. 6.*

The good effect, though only for a short while, may provide some patients with chronic pain a motivation to try other treatments to see if it would last longer the next time, but as in this case, it may also create frustration when numerous attempts to reduce the illness seem futile.

### Familiarity

When participants were asked about their experiences with the Danish healthcare system and the differences compared with the healthcare system(s) in their region of origin, they emphasised the importance of feeling comfortable and safe in the healthcare system. Language and communication in the meeting with healthcare professionals was most often the main focus when describing what made them feel comfortable and safe. Most of the participants who had tried utilising healthcare services in their region of origin explained that language barriers and communication problems were not the main reasons for their use of healthcare abroad. However, when asked about communication and comprehension in Denmark and in the region of origin, many felt it was easier and more straightforward to speak with a doctor in their region of origin, in their own language. This was of course influenced by some of the participants’ limited Danish language skills. Most participants felt they had access to an interpreter whenever they needed one during a meeting with healthcare professionals in Denmark. A participant described having difficulties in communicating through the interpreters and expressed that despite having insufficient Danish language skills, she preferred speaking with doctors on her own:

*‘If I can speak on my own, face-to-face, it’s easier to understand one another. You can feel each other in a way, without an interpreter. So, yes, I don’t speak fluently, but I can feel more if you talk face to face.’ – Participant no. 10.*

Most participants who spoke Danish at some kind of basic level expressed this opinion. But very few participants could speak Danish at an adequate level, and to those an interpreter was the only way to communicate with the healthcare professionals. However, many participants expressed a dislike of speaking through an interpreter because they felt a lot of meaning and information was lost in the translation. Thus, many participants explained how speaking to a doctor who spoke their language made it much easier to have satisfying communication. One participant expressed it as follows:

*‘If you can speak in your own language, you can explain so many things, like what you feel, and you can explain the medical things, unlike now where you have to think: what’s the meaning of this and that? In your own language, you can use more complicated words and explain yourself and your condition better to the doctor. If you don’t speak fluently, it can be difficult.’ – Participant no. 10.*

### Affordability

In order to receive the services abroad, the participants had to pay out-of-pocket, as they were not insured abroad. Whether or not participants seek healthcare in their region of origin were therefore also perceived to be a question of affordability, as explained by a participant who had not tried seeking healthcare services in her country of origin due to economic constraints but had many friends and relatives who had:

*‘Some people I know, they’ve been to Turkey and some of them got good treatment, much better hospital and medicine and much faster. In two hours, they visited a private hospital and they examined them bottom-up. They’re very content, but then again, it’s expensive.’ – Participant no. 7.*

Time appeared to be a key element for the patients, both during treatment and when seeking help, and time and money seem to go hand in hand, as one participant explained:

*‘In Lebanon, if you want to visit a specialist, you can just go because you can pay. You can come in the middle of the night and wake them up. It’s okay because you pay them. Here, you’ll have to wait a looong time, wait, wait, wait.’ – Participant no. 10.*

This illustrates how the participant sees a relationship between, on one hand, time and availability and, on the other, value and quality. Getting treatment fast appears as valuable and an indicator of good quality. This may explain why patients find the quality of the healthcare service in their region of origin better and therefore worth paying for.

## Discussion

This study investigates the motives for using healthcare services in the region of origin among 10 non-Western immigrants living in Denmark. Our study thereby presents the motives and perspectives of a vulnerable group of patients, which so far have not been investigated in a Scandinavian context. Other studies have found that cross-border healthcare use was mostly seen among patients with good economic resources [18, 20, 22] or, on the contrary, among patients who did not have health insurance in their country of residence and/or to whom the cross-border healthcare use was more affordable [18, 25, 27]. Since the group of patients we included did not belong to either of these groups, this study involves groups so far not investigated and reveals new knowledge about motives, including the importance of affordability.

In the present study, the most important motives for cross-border healthcare use were found to be the perceived availability and quality of services as well as issues related to familiarity (including language proficiency) and seeking a second opinion. Furthermore, the participants mentioned affordability of healthcare services as a factor influencing the availability, the quality, and the need for a ‘second opinion.’ In the following, these different factors, their significance, and their interplay in shaping cross-border healthcare use will be discussed.

The participants described lack of availability of services in Denmark as one of the most important motives for seeking healthcare in their region of origin. They were dissatisfied with the long waiting times caused by the referral system where GPs act as central gatekeepers to more specialized healthcare services, and by experiences of what they described as inefficiency among Danish doctors. According to Glinos et al., availability of healthcare services can be limited due to inadequate quantities of services and structural barriers [18]. As long waiting times can be seen as an indicator of an inadequate quantity of services and the Danish referral system can be regarded as causing structural barriers, our findings support this typology. Availability had a dual character of both being a push and a pull factor since the general practitioners acts as gatekeepers and restrict access to specialized healthcare services in Denmark, which is a push factor. This is in contrast to the health care systems in the region of origin of our participants, where the direct out-of-pocket payment often gives direct access to specialized care, without gate-keepers or need for referrals [28]. We consider this to be a pull factor.

It is likely that some non-immigrant, ethnic Danish patients can be dissatisfied with the availability of services in the Danish healthcare system in the same way as immigrant patients. However, the considerably higher use of cross-border healthcare services among immigrant patients than among ethnic Danes indicates that immigrant patients find other alternatives to the Danish healthcare services than non-immigrant, ethnic Danes do [16].

The perceived quality of healthcare services in Denmark and in the region of origin was found to be another important motive when participants decided where to seek healthcare assistance. To some participants, a high quality of healthcare services was associated with easy access in particular to specialised treatment. To others, the main focus was the length of the time they had to wait for results, clear information on the treatment plan, or the effect of the treatment they were given. The latter is in line with findings by Sekercan et al. (2014) who also found that dissatisfaction with the quality of the care received in the Netherlands was an important motivation for seeking healthcare in the country of origin, especially among people with Turkish origin [17].

The wish for a second opinion from healthcare professionals in the region of origin was described as a motive by some of our participants and is also mentioned as an important motive by Sekercan et al. [17]. In the present study, we found that seeking a second opinion for some was a natural part of the participants’ healthcare seeking behaviour. Others described how they were motivated by a feeling of desperation, which occurred when there were no more treatment options in the Danish healthcare system. The fact that many female patients with an immigrant background make repeated visits with the same chronic illness to which the doctors, after thorough examinations and referrals, have no more treatments to offer is described in a report made by the Danish Institute of Human Rights [29]. The wish for a second opinion could be an indicator of the patient feeling uncertain which may be partly tied to miscommunication in the encounter between the immigrant patient and the Danish healthcare system. However, it could also be acknowledged as an example of active healthseeking behaviours among patients who consider themselves the main actors regarding their health and illness, not leaving the full responsibility in the hands of the Danish doctors. This is in line with a general development in patient-provider roles, where patients are active decision makers and equal partners in cooperation with their doctors [30, 31].

Consistent with previous research [14, 15], we found examples of participants who disagree with the examination and treatment they have been given in Denmark and participants who prefer a higher involvement in the decision making than they have [32]. This shows a contradiction between healthcare professionals and the immigrant patients regarding the role and behaviour of the patient, whether active or passive in decision making and not least when a patient is considered active and cooperative [8, 33].

Language proficiency and familiarity seemed to affect the participants’ expectations of the health care system and play an important role in whether they felt included or excluded from decision-making. Familiarity is among other things shaped by culture, ethnicity, socio-economic factors and level of acculturation [6, 7, 18]. Other studies have also found familiarity, including language and communication, to be one of the most important motives for seeking healthcare abroad [22, 24]. In the present study none of the participants described familiarity or language barriers as the main reason for seeking healthcare abroad, but many described it as a push factor in Denmark and a pull factor for seeking healthcare in the region of origin. However, it is difficult to speak of the influence of familiarity as an isolated motive, as it is a factor that can very likely influence the perception of quality and availability. Being in a familiar environment with the possibility of speaking with health professionals in the patients’ own language can influence not only the perception of quality and access but also the clinical diagnosis and treatment of the patient [33, 34].

Affordability and economic resources can have an effect on the other motives for seeking healthcare abroad, as good economic resources influence the availability, obtainable quality, and the possibilities for a second opinion. The importance of affordability as a motive may therefore be affected by the number of push and pull factors. If there are many push and/or pull factors, affordability becomes less important, though it will naturally limit the use of healthcare. This could be an explanation for why affordability was mentioned more by those of the participants who felt they did not have the economic resources to use healthcare services in their country of origin. Those who had the resources often stated that they knew it could be expensive, but as it was related to their health and the quality of the services was good, they felt it was worth the money. As affordability did not emerge as an individual reason to seek healthcare abroad but only as a reason for not seeking healthcare abroad, it is not considered a push or pull factor in this study. None of the participants mentioned that they had considered using a private hospital in Denmark. Had they done so, economy would probably have been described as a push factor, as healthcare services in the Danish private sector in all probability are more expensive than in the private healthcare sector abroad. Our findings on economic motives are in contrast to the findings by Migge and Gilmartin (2011), who found affordability to be one of the main reasons for immigrants to seek healthcare in their country of origin [23, 24]. The different results might be explained by structural differences between national healthcare systems in Ireland and Denmark, where the amount of co-payment and the need for health insurances may be important push factors, though other factors, like health literacy in general and familiarity with the healthcare system in the host country among the immigrant population, could also be relevant explanations.

### Methodological reflections

In qualitative research, it is important to reflect on the context of the research, including recruitment of participants, the interview setting, and the contribution of researchers to the data constructed during interviews and the results emerging through the analysis of these data [35]. The participants were all recruited through a medical clinic designed specifically to meet the needs of immigrant patients with complex and sometimes longstanding health problems. The patients at this clinic are likely to be experiencing health problems that the mainstream healthcare services have not been able to solve in a satisfactory manner, and they are thus more likely to have experiences with the subject matter of this study. While this is an advantage due to the access it gives us to the experiences of patients, it is important to note that the experiences of these patients are not necessarily reflective of experiences among immigrant patients with less complex health issues. The most vulnerable patients were screened out before recruitment was initiated to avoid burdening patients with severe physical or mental health conditions. Further, as we only included women, we are unable to include gender perspectives in our findings. The study population is not representative of patients in the immigrant clinic in terms of gender, but it does reflect the general composition of patients in the clinic with regard to ethnicity, age, and occupational status.

The interview setting enabled convenient access to a sample of immigrant patients. Nevertheless, conducting interviews in a clinical setting may cause participants to be reluctant to speak more openly on negative experiences with the Danish healthcare system during the interview [36]. In the interview situation, we tried to counter this by signalling that the researcher was not associated with the clinic by wearing clothe that were different to what the staff wore, offering hot beverages to the participants, and giving the participants a symbolic gift afterwards, thereby creating a distance to the hospital atmosphere. We tried to make the aim of the interview clear to our participants, especially the fact that their answers and experiences would not be shared with the staff at the clinic. Furthermore, as the main focus of the interview was the participants’ perceptions and experiences, the questions about exact information on when, how, and who were involved in negative encounters with healthcare services, which could make the interview seem interrogative, were avoided. Thus, we might have reduced some of the potential negative effects of the setting, although we fully acknowledge that conducting interviews in informal settings, such as the participants’ homes, would have added more contextual information and perhaps more critical perspectives on healthcare use.

During analysis, results were discussed within the group of researchers involved in the study as well as with a group of interdisciplinary migrant health researchers. Through this process of reflexivity, actively addressing preconceptions, challenging analytical terms, and focusing on both commonalities and differences in experiences across patient cases, the validity of analysis was strengthened.

As mentioned earlier, using interpreters in qualitative studies raises a number of ethical and methodological concerns (e.g., the participants and their answers can be influenced by the interpreter and information can be lost because of inaccurate translation). However, using an interpreter is necessary if research is to include perspectives of immigrants with less language fluency. We tried to diminish the negative effects of using interpreters by using short sentences with only one question at a time and using simple and nonmedical words [37]. The clinic often involves the same interpreters, preferably those they have had good experiences with beforehand. In our interview setting, the participant and the interpreter were therefore sometimes familiar with each other, which seemed to make the participant feel more comfortable. A possible advantage of using video interpreters is that it makes the interpreter less physically present, leaving the participant and the interviewer in a more private atmosphere thus facilitating a more direct, intimate interview situation. On the other hand, the lack of presence of an interpreter restricts possibilities for small-talk before and after the interview.

Our study population represents a vulnerable group of immigrant patients with a high prevalence of disease. If the use of healthcare services abroad is influenced by the perception of need for healthcare and the resources available for seeking healthcare abroad, our study population might have a higher perception of need for healthcare services than the general immigrant population because of their high morbidity. However, they might have fewer resources available for seeking the healthcare services abroad. Therefore, future quantitative studies are needed to determine the extent of cross-border healthcare use among immigrants in Western societies as well as the relative importance of the motives identified in our study. The perception of immigrants’ use of healthcare services abroad among health professionals would also be an interesting area of investigation, asking why, according to the health professionals, immigrants use healthcare services abroad and how the health professionals handle this when they are confronted with it in their daily work.

## Conclusion

This study has investigated the motives for seeking healthcare services in the region of origin by interviewing 10 patients with an immigrant background. We found availability, perception of quality, familiarity, and the need for a second opinion to be the most important motives. We thought it relevant to assess the motives as push and pull factors because these factors exist both in the country of residence and in the region of origin. Affordability was an influence on the other motives but was not a motive on its own. The motives leading immigrant patients to seek healthcare services abroad reflects a conflict between patients with an immigrant background and healthcare professionals in terms of perception of the patient role and expectations toward the healthcare system. Further research, both quantitative and qualitative, should be initiated to investigate the use of cross-border healthcare among people with an immigrant background including research on the relative importance of the motives and the perspective of the healthcare professionals.

## Additional file

Additional file 1: Semi-structured Interview guide. (DOCX 20 kb)

## Abbreviations

MRI: magnetic resonance imaging
