# Supplementary material for: Use of healthcare services in the region of origin among patients with an immigrant background in Denmark: a qualitative study of the motives
Source: BMC Health Serv Res. 2016 Mar 21;16:99. doi: 10.1186/s12913-016-1346-1 (PMC4802714; doi:10.1186/s12913-016-1346-1)
Supplement: Additional file 1: — Semi-structured Interview guide. (DOCX 20 kb) [file 12913_2016_1346_MOESM1_ESM.docx]

**Semi-structured Interview guide**

**Introduction**

*My name is Nicoline and I am undertaking a study which examines the use of different kinds of medication among patients at this clinic.*

*The aim of the study is to improve the treatment and delivery of services to patients at this clinic.*

*Since you are a patient here, you have important knowledge about your treatment, which can contribute to the study.*

*There are no right or wrong answers in this, you are the expert and everything you will tell me will be kept confidential and anonymous.*

Introduction to the Dictaphone*: If it is okay, I would like to record our conversation? The recordings will also be kept confidential.*

*I will now read aloud the written consent and afterward ask you to sign it if you can agree to this.*

*- Do you have any questions before we begin?*

**Introductory questions**

*Now I would like to ask you some general questions about your background and your experience with the Danish healthcare system. Then, I will ask you some questions about your use of healthcare services abroad.*

- How old are you?
- Do you have a family, and do you live with this family in Denmark?
- Where did you grow up? Where do you come from?
- How long have you lived in Denmark?
- Have you been working while you have been in Denmark?
- Did you work before you came to Denmark?
- Have you taken any education in Denmark or elsewhere?

**The participants perception of own health**

Now I would like to ask some questions about your health and your medical treatments.

- Can you describe your illness to me?
- Can you describe the treatment you receive at the moment to me?
- How does your health status affect your everyday-life?
- Do you take any medicine?
- Can you describe the kind of medicine you receive?
- Do you have anyone, either in your family or among friends, who you talk to about your health?
- Do you feel the treatment you receive improve your illness?

**Health related issues**

*I will now ask you some questions about issues, which I know many other patients have. I am interested in whether you have the same kind of issues and I would like you to tell me about it. If you don’t feel you have these issues, it is okay to say you don’t.*

**Perceived health**

- Have you felt, either here at the clinic or other places in the Danish healthcare system that you were uncertain of or in disagreement with the doctor’s opinion on your health problems?
- Have you ever felt that you were given a treatment (e.g. medicine) for reasons you did not understand?

**Communication with the Danish healthcare system**

*Some patients experience having problems in communicating with the Danish healthcare system…*

- Have you experienced difficulties with understanding the healthcare professionals (doctors, nurses, etc.) or felt misunderstood, either here or in other parts of the Danish healthcare system?
- (If yes) Will you describe this situation to me?
- What do you normally do if you feel misunderstood?

**Healthcare services**

*Now I would like to talk about your experience with receiving healthcare services in Denmark*

- Have you ever felt uncertain about *how* to take you medication?
- Have you ever felt that the medicine you were given had another effect than you expected? (If the participant answers “yes”: how?)
- Have you ever avoided or failed to follow a treatment, e.g. medication, prescribed to you by a health professional?
- If yes, will you describe the situation to me?
- Besides medication, would you do something else if you feel ill or have symptoms?

**Healthcare services in region of origin**

*I’ve heard about other patients, who go back to their country or region of origin to get healthcare services. I would like to learn more about this*

- Do you have any experience with this? Or do you know anyone who has?
- Since you moved to Denmark, have you ever tried receiving a healthcare service in your country or region of origin (e.g. seeing a doctor, being scanned or receiving advice on medication)? E.g. during a holiday?

**If “yes”:**

- Can you describe the healthcare services you received in your region of origin? (When or how?)
- Can you describe to me why you chose to get the healthcare service?
- Have you tried taking other actions while you were there to improve your health?
- Did someone (e.g. friends or family) help you make contact with the healthcare system in your region of origin?
- Have you received treatments in your region of origin, which you normally can’t get in Denmark?
- What do you perceive to be the difference between the treatment you received in your region of origin compared to the treatment you received in Denmark? Do you consider it better or worse?
- Was it more or less easy to understand the treatment you were given in your region of origin?
- How did the treatment you received in your region of origin affect the treatment you received when you returned to the Danish healthcare system?
- When you had received healthcare services in your region of origin, did you feel you could talk to your doctor in Denmark about this experience?
- Have you tried talking to your doctor in Denmark about the medication or healthcare services you’ve had in your region of origin?

**If the participant answers “no” when asked about experiences with healthcare services in the region of origin:**

- Do you think there are any differences between the healthcare system in your country or region of origin and the healthcare system in Denmark?
- Do you ever use any traditional treatments from your region of origin if you feel ill? (e.g. herbs, tea, alternative therapies etc.)

**Closure**

*I have no more questions*

- Do you have anything you would like to ask me about?

*Thank you very much for taking your time to participate.*
